# Clinical relevance of different biomarkers in imported plasmodium falciparum malaria in adults: a case control study

**DOI:** 10.1186/1475-2875-12-246

**Published:** 2013-07-16

**Authors:** Sabine Stauga, Andreas Hahn, Norbert W Brattig, Johanna Fischer-Herr, Stephan Baldus, Gerd D Burchard, Jakob P Cramer

**Affiliations:** 1 Section Tropical Medicine, I. Department of Internal Medicine, University Medical Center Hamburg-Eppendorf, Hamburg, Germany; 2 Clinical Research Group, Clinical Research & Epidemiology Section, Bernhard Nocht Institute for Tropical Medicine, Hamburg, Germany; 3 Molecular Medicine Department, Clinical Research & Epidemiology Section, Bernhard Nocht Institute for Tropical Medicine, Hamburg, Germany; 4 Department of General and Interventional Cardiology and Cardiovascular Research Center, University Heart Center, Hamburg, Germany

**Keywords:** Malaria, Plasmodium falciparum, Complicated malaria, Biomarkers, Prognostic biomarkers

## Abstract

**Background:**

For rapid initiation of anti-malarial treatment and prevention of complications, early diagnosis and risk stratification is important in patients with Plasmodium falciparum malaria. Routine laboratory values do not correlate well with disease severity. The aim of this study was to determine the diagnostic and prognostic value of several biomarkers related to inflammation; endothelial and cardiac dysfunction; coagulation, and haemolysis in imported P. falciparum malaria.

**Methods:**

In a prospective case-control study, 79 adult travellers with both uncomplicated and complicated P. falciparum malaria were included between 2007 and 2011. Forty-one healthy subjects were included as controls. Blood samples were obtained within 24 hours after first consultation to assess routine laboratory values as well as markers related to inflammation (PAPP-A, copeptin, CRP), endothelial activation (MPO, elastase-2, endothelin-1, sICAM-1, sVCAM-1), cardiac function (NT-proBNP, MR-proANP), coagulation (fibrinogen, D-dimers, platelet count), and haemolysis (LDH). Prognostic performance was assessed using the receiver operating characteristic curve (area under the curve = AUROC).

**Results:**

Twelve (15.2%) patients had severe P. falciparum malaria. In the patient group, significant thrombocytopaenia was found, all other markers but PAPP-A were significantly elevated. Diagnostic performance was best for CRP with an AUROC of 1.00, followed by MPO (0.99), D-dimers (0.98), elastase-2 (0.98), and sICAM-1 (0.98). Biomarker levels did not correlate well with disease severity.

**Conclusion:**

The combination of travel history, fever prior to blood sampling, and CRP serum levels above or below 10.8 mg/l upon hospital admission, best discriminated between malaria patients and control persons. None of the biomarkers studied predicted the presence or the development of malaria complications, neither at the time of admission, nor during hospitalization.

## Background

Malaria remains the most important parasitic disease with an estimated number of deaths of 1.2 million in 2010, especially amongst children under five [1,2]. More than 125 million travellers visit malaria-endemic regions each year and more than 10,000 return with malaria [3]. Plasmodium falciparum malaria is a medical emergency [4]. For rapid initiation of anti-malarial treatment, and in order to prevent complications, early diagnosis is important, in particular with non-immune travellers. Molecular genetic techniques have added diagnostic sensitivity and species specificity to classic blood microscopy, but they remain both expensive and time consuming.

Prompt anti-parasitic treatment does not always prevent complicated courses of disease. Clinical symptoms can deteriorate rapidly, progressing to life-threatening conditions, such as coma, renal failure and pulmonary oedema [5,6]. The onset of complicated malaria is not always reflected by significant changes in standard laboratory and parasitological parameters. For example, the progression from mildly impaired consciousness to coma, may occur within hours at low peripheral parasite levels, and with normal or slightly altered biochemical laboratory parameters. Prognostic markers could be of clinical relevance as case fatality rates rise rapidly with time and can reach up to 10.5% in imported severe malaria [7]. In industrialized countries, many malaria patients initially present to physicians who, lack expertise in diagnosing Plasmodium infection, judging the disease severity, and prognosis after the diagnosis has been established by standard microscopy. Diagnosis and treatment may be postponed until the patient is referred to a tropical disease specialist. In this setting it would be favourable to have a combination of biomarkers that are sensitive for malaria and correlate with disease severity.

Biomarkers are distinct biochemical substances that are indicators of a patient’s current state of health. They can be used to diagnose and predict the progress and outcome of many diseases. Ideally, a biomarker should fulfil the following criteria: i) ability to diagnose a disease; ii) ability to identify patients at risk; iii) ability to stratify patients depending on disease severity; iv) ability to provide prognosis; v) ability to provide guidance in treatment; and, vi) ability to identify the risk for long-term complications.

The parasite’s virulence factors, as well as the host’s immune system, play an important role in the disease’s clinical manifestation and severity. Plasmodium falciparum malaria is a multisystem disorder, potentially affecting every organ of the body. Due to P. falciparum-specific pathophysiology, systemic inflammation, endothelial activation, coagulopathy, and circulatory dysfunction often occur.

A panel of markers related to inflammation [proarginin-vasopressin (copeptin), metzincin pregnancy associated plasma protein-A (PAPP-A), C-reactive protein (CRP)], endothelial activation [myeloperoxidase (MPO), elastase-2, endothelin-1, soluble intercellular adhesion molecule-1 (sICAM-1), soluble vascular adhesion molecule-1 (sVCAM-1)], cardiac function [N-terminal pro-brain natriuretic peptide (NT-proBNP), midregional pro-atrial natriuretic peptide (MR-proANP)], and coagulation (fibrinogen, D-dimers, platelet count) was selected. Lactate dehydrogenase (LDH) was determined as a marker for haemolysis. PAPP-A and myeloperoxidase had not been tested in malaria before.

Regarding the role of these parameters, two aspects were examined in this case control study: i) the potential of diagnostic capacities, or whether there is an association with imported P. falciparum infection at all; and, ii) the association with disease severity. The hypothesis that particularly arises in this context is that if the biomarkers correlate with disease severity in P. falciparum malaria, according to World Health Organization (WHO) criteria or to national guidelines, patients with elevated markers are at risk even without consideration of the guidelines. However, to prove this hypothesis further prospective studies are needed.

## Methods

### Study site and participants

Between June 2007 and February 2011, patients with imported P. falciparum malaria were screened at the in- and outpatient departments of the Section for Tropical Medicine at the University Medical Centre Hamburg-Eppendorf, which is a tertiary hospital in Hamburg, Germany. Eligible patients had to be older than 18 years. Exclusion criteria were evidence of other significant infection, inability to give informed consent, and pregnancy. Complicated malaria was diagnosed according to the current guidelines of the German Society for Tropical Medicine and International Health (DTG) and modified WHO criteria [8,9] (see Additional file 1). In brief, criteria for severe malaria included acute renal insufficiency (serum creatinine >2.5 mg/dl), hyperparasitaemia (>5% parasitized erythrocytes), severe anaemia (haemoglobin <80 g/l), and neurological symptoms (impaired consciousness, coma or cerebral convulsions). Anti-malarial treatment was initiated according to the above-mentioned national guidelines and consisted mainly of mefloquine or atovaquone/proguanil for uncomplicated, as well as intravenous quinine in combination with either doxycycline or clindamycin for complicated life-threatening disease. Healthy adult volunteers were screened as controls.

### Study procedures

At the first presentation, physical examination was performed by a study physician after obtaining informed consent and taking the history. Clinical parameters included arterial blood pressure, heart rate and axillary body temperature. Plasmodium falciparum infection and parasite density were diagnosed by conventional light microscopy of asexual blood stage parasites from Giemsa-stained thick and thin blood smears. A parasitaemia <1% was calculated from thick film, according to the number of leukocytes per 20-50 fields of vision. If parasitaemia was ≥1%, parasites were calculated from thin films, according to 300-1,000 erythrocytes. Within the first 24 hours of presentation routine blood parameters, including full blood count, creatinine, liver enzymes, and total bilirubin were assessed and additional samples were obtained for the inflammatory markers copeptin, PAPP-A and CRP. The endothelial markers tested were MPO, elastase-2, endothelin-1, sICAM-1, and sVCAM-1. The cardiac markers tested were NT-proBNP and MR-proANP. Further markers included the coagulation markers fibrinogen und D-dimers, and LDH as a reference marker for haemolysis.

CRP, LDH, differential full blood count, fibrinogen and D-dimers were measured by standard in-house tests. The following test kits were used for the remaining, above mentioned biomarkers: BRAHMS Copeptin KRYPTOR, PAPP-A KRYPTOR, and MR-proANP KRYPTOR (commercially available immunofluorescence assays by Thermo Scientific, Hennigsdorf, Germany, who kindly provided some of these kits), ELH-sICAM1-001, (Human ELISA kit by RayBio, Norcross, USA), Human sVCAM-1 Platinum ELISA BMS232 (by eBioscience, Frankfurt, Germany), PMN Elastase kit BE59311 (ELISA by IBL-America Minnesota, USA), endothelin kit BI-20052 (ELISA by Biomedica, Vienna, Austria), CardioMPO kit (by Cleveland HeartLab 7601, Ohio, USA). The NT-proBNP ELISA used was Dimension Vista System by Siemens Healthcare Diagnostics Ltd, Camberley, UK. The commercial kits were used according to the protocols of the various manufacturers who ensured quality control and reproducibility. Reproducibility was verified by selected replicates, quantification was achieved by standard curves performed in duplicates. Blinded testing of the samples was performed. In order to assure the good quality of biomarker research, study design and procedures were conducted according to the Reporting Recommendations for Tumor Marker Prognostic Studies (REMARK) checklist [10].

### Statistical analysis

SPSS statistics 17.0 software (SPSS Inc.^®^ Chicago, IL, USA) was used for statistical analyses. In order to obtain information about the biomarkers’ potential for differentiation between malaria patients and controls, as well as between patients with uncomplicated and complicated malaria, results were graphically displayed in box plots. Next, the biomarkers were examined applying receiver operating characteristic (ROC) curves for illustration of sensitivity and specificity. The area under the ROC curve (AUROC) and their corresponding 95% confidence intervals were calculated. The highest AUROC possible was 1.0, defining the theoretically most perfect test with a sensitivity and specificity of 100%. An AUROC of 0.8-0.9 represented a good test, an area of 0.7-0.8 a fair test. A result <0.7 was considered a poor test, an area of 0.5 indicated a worthless test. Pearson’s correlation co-efficient was used to assess correlation amongst the different biomarkers. No significant association was found between test results and creatinine levels. Each marker’s best cut-off point was calculated by Youden’s index. A P-value of <0.05 was set as level for statistical significance. Due to the pronounced high collinearity, multivariate analysis was not done.

### Ethic approval

The study protocol was approved by the Ethics Committee of the Board of Physicians in Hamburg, Germany.

## Results

### Baseline characteristics

Of 91 patients with imported P. falciparum malaria initially screened, 79 were found to be eligible according to the study protocol and were included in the study. Four female and six male patients were excluded from the study because of accompanying significant infection, such as hepatitis B (n = 4), HIV (n = 3), Herpes simplex viral pneumonia (n = 1), adnexitis (n = 1) and malignancy, i e, prostate cancer (n = 1). Two women were excluded because of pregnancy. Fifty-four (68.4%) and two (2.5%) of the patients were migrants from Africa and Asia, respectively, and twenty-three (29.1%) were Caucasian. The majority of patients (96.2%) acquired P. falciparum malaria in Africa, only one (1.3%) infection was acquired in Central America. A total of four (5.3%) patients reported to have taken adequate malaria prophylaxis: one patient with complicated and two patients with uncomplicated malaria had taken mefloquine, one patient with uncomplicated disease had taken atovaquone/proguanil. More than half of all malaria patients (64.8%) developed symptoms within the first seven days after returning from their journey, almost one quarter (23.0%) within seven to 14 days, and 5.4% and 6.8% within 14 to 21 days and >21 days, respectively. Nine (81.8%) of the patients with complicated disease developed symptoms within the first week after returning from their travels. Twelve (15.2%) patients were categorized as complicated malaria for the following reasons: parasitaemia >5% (n = 10), severe anaemia (n = 1), neurological symptoms (n = 4), and renal failure (n = 2). Four of the patients presented with more than one criterion defining complicated disease. None of the patients died. All 41 healthy controls were Caucasians from Germany. The mean age was 33.51 years (age range 18-72 years), twenty-four subjects (59%) were female.

The heart rate and body temperature was significantly higher in the patient group compared with the control group: mean heart rate was 90 bpm versus 70 bpm (P <0.001) and mean body temperature was 37.8°C versus 36.5°C (P <0.001), respectively. The mean systolic arterial blood pressure was similar in malaria patients and controls with a value of 119.6 mm Hg and 122.4 mm Hg, respectively (P = 0.283). The geometric mean parasite density (GMPD) was 17,349/μl in all malaria patients, and 255,946/μl and 10,635/μl (P <0.001) in complicated and uncomplicated cases, respectively. The mean/median levels of standard laboratory parameters characteristic for malaria like haemoglobin, leukocytes, platelets, as well as liver enzymes, total bilirubin and serum creatinine were significantly altered in the malaria patient group when compared with the healthy controls. Both the demographic and clinical characteristics, as well as basic laboratory results, are shown in Table 1.

**Table 1:** **Demographic**, **clinical and biochemical characteristics of Plasmodium falciparum malaria cases at first presentation and healthy controls**

	**Malaria (n=79)**	**CM (n=12)**	**UM (n=67)**	**Controls (n=41)**	P-value*
Gender, male / female	60 / 19	7 / 5	53 / 14	17 / 24	< 0.001
Age	43.8	51.7	42.4	32.5	< 0.001
(range)	(21–69)	(29–69)	(21–65)	(18–72)	
Travel destination (n/%)					
Africa	76 (96.2)	11 (100.0)	65 (98.5)	NA	NA
South America	1 (1.3)	0	1 (1.5)		
Duration of stay, days (%)					
< 7	4 (5.5)	0	4 (6.25)	NA	NA
7 - 14	9 (12.3)	2 (22.2)	7 (10.9)		
14 - 21	7 (9.6)	2 (22.2)	5 (7.8)		
> 21	53 (72.6)	5 (55.6)	48 (75.0)		
Time to development of complaints after travel, days (%)					
< 7	48 (64.8)	9 (81.8)	39 (61.9)	NA	NA
7 - 14	17 (23.0)	2 (18.2)	15 (23.8)		
14 - 21	4 (5.4)	0	4 (6.3)		
> 21	5 (6.8)	0	5 (7.9)		
Malaria prophylaxis, n (%)	4 (5.3)	1 (9.1)	3 (4.6)	NA	NA
Temperature, °C (±SD)	37.8 (1.2)	37.9 (1.2)	37.8 (1.2)	36.5(0.4)	< 0.001
BP, mmHg (±SD)	119.6 (16.0)	118.9 (11.8)	119.7 (16.7)	122.4 (11.7)	0.283
Heart rate, bpm (±SD)	90 (18)	92.5 (18.5)	89.6 (17.6)	70 (8.4)	< 0.001
Haemoglobin, g/l (±SD)	131.6 (19.3)	121.4 (25.0)	133.5 (17.7)	137.8 (11.0)	0.030
Platelets, x1,000/μl (±SD)	96.6 (63.0)	52.6 (33.4)	104.4 (63.9)	250.2 (87.1)	< 0.001
Leukocytes, x1,000/μl (±SD)	5.5 (2.3)	5.8 X(2.3)	5.4 (2.3)	6.3 (1.1)	0.014
Parasite density, parasites/μl (±SD)	97,974 (176,183)	407,028 (274,253)	41,783 (56,595)	NA	< 0.001
Total bilirubin, mg/dl (±SD)	1.7 (1.2)	2.6 (1.6)	1.5 (1.1)	0,7 (0.5)	< 0.001
ALT, U/l (±SD)	44.9 (30.7)	68.0 (45.9)	40.8 (25.4)	23,8 (13.2)	< 0.001
AST, U/l (±SD)	51.8 (50.6)	108.6 (105.9)	41.7 (21.6)	25.3 (5.9)	< 0.001
Creatinine, mg/dl (±SD)	1.1 (0.4)	1.6 (0.8)	1.1 (0.2)	0.8 (0.1)	< 0.001

Mean (+SD) if not indicated otherwise; BP = systolic blood pressure; NA = not applicable; SD = standard deviation; Malaria = all P. falciparum malaria patients; CM = complicated malaria; UM = uncomplicated malaria; *Comparison between all malaria patients and healthy controls (t-test).

### Biomarkers analysed in malaria patients versus controls

**Figure 1:**
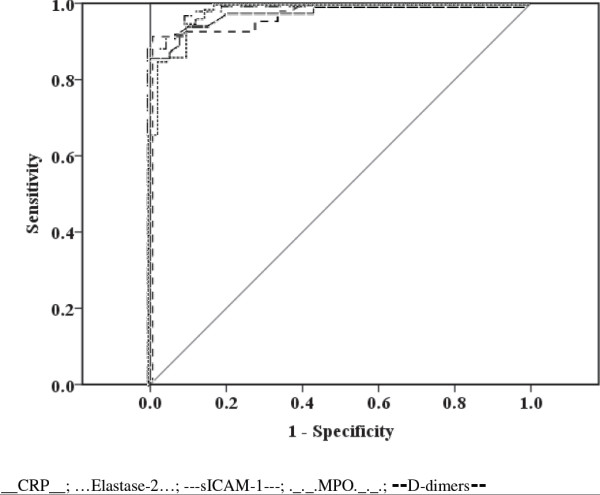
**Receiver operating curves (ROC), diagnostic performance of CRP, elastase-2,sICAM-1, MPO, D-dimers for Plasmodium falciparum malaria.**

The following serum levels were significantly higher in the patient group when compared with the healthy controls: copeptin, CRP, MPO, elastase-2, endothelin-1, sICAM-1, sVCAM-1, NT-proBNP, MR-proANP, fibrinogen, D-dimers, and LDH. Median values for PAPP-A were similar in both groups. The diagnostic performance and accuracy was measured by AUROC and the corresponding 95% confidence intervals (95% CI). Five biomarkers showed the highest AUROC in all malaria patients compared to the control group: CRP (1.00), MPO (0.99), D-dimers (0.98), elastase-2 (0.98), and sICAM-1 (0.98) (see Figure 1). Results for LDH, platelets, sVCAM-1, copeptin, and fibrinogen were slightly lower than for the markers mentioned above. AUROC of endothelin-1, NT-proBNP, and MR-proANP were inferior to the above. Additional files 2 and 3 summarize the results of all measured biomarkers in all malaria patients and healthy controls.

### Biomarkers analysed in complicated malaria versus uncomplicated malaria

Median values of CRP, MPO, sICAM-1, sVCAM-1, D-dimers, platelets, and LDH differed significantly between the two groups. However further analysis via receiver operating curve showed large 95% CI, indicating poor association with disease severity for all markers. No significant association was found for copeptin, elastase-2, or fibrinogen. Table 2 and Additional file 3 show the results of the above-mentioned biomarkers in both complicated and uncomplicated malaria.

**Table 2:** **Biomarkers in complicated malaria** (**CM**) **versus uncomplicated malaria** (**UM**), **measured within 24 hours after first presentation**

	**CM / UM**	**Number (n)**	**Median value**	**AUROC**	P-value*	**95% CI**	
**Inflammatory markers**							
Copeptin (pmol/l)	CM	12	12.5	0.596	0.293	0.420	0.771
	UM	67	8.9				
CRP (mg/l)	CM	12	165.5	0.771	0.003	0.645	0.896
	UM	67	78.0				
**Endothelial markers**							
MPO (pmol/l)	CM	12	4747.8	0.699	0.029	0.569	0.829
	UM	67	2827.1				
Elastase-2 (ng/ml)	CM	12	2850.5	0.562	0.495	0.416	0.708
	UM	67	3227.0				
sICAM-1 (ng/ml)	CM	12	257.0	0.784	0.003	0.650	0.919
	UM	67	136.4				
sVCAM-1 (ng/ml)	CM	12	1657.5	0.752	0.006	0.604	0.900
	UM	67	1020.0				
**Coagulation markers**							
Fibrinogen (g/l)	CM	12	4.7	0.482	0.842	0.287	0.676
	UM	59	5.0				
D-dimers (mg/l)	CM	7	3.7	0.775	0.019	0.643	0.915
	UM	51	1.4				
Platelets (x1000/μl)	CM	12	51.0	0.777	0.002	0.646	0.908
	UM	67	91.0				
**Haemolysis marker**							
LDH (U/l)	CM	12	385.0	0.810	< 0.001	0.697	0.924
	UM	65	260.0				
GMPD (parasites/μl)	CM	12	255,946	0.926	< 0.001	0.826	1.000
	UM	66	10,635				

Only showing markers that distinguish well between malaria patients and healthy controls 95% CI = 95% Confidence Interval for AUROC; GMPD = geometric mean parasite density.

*P-value for AUROC.

## Discussion

The aim of this pilot study was to assess the diagnostic performance and discriminative capacity of novel, as well as established biomarkers, in imported P. falciparum malaria, measured within the 24 hours after first presentation. CRP serum levels above or below 10.8 mg/l upon hospital admission, in combination with travel history to an endemic area, and fever during the days prior to blood sampling, best discriminated between malaria patients and control subjects. Several of the other biomarkers tested also discriminated well between malaria patients and control persons. However, neither at the time of first presentation, nor during the course of the hospital stay did any of the biomarkers studied here predict the presence, or the development of, complicated malaria.

The results reflect the heterogenicity of pathophysiological mechanisms in P. falciparum malaria according to WHO criteria. As in sepsis systemic inflammation and endothelial activation play a major role in severe malaria, explaining why almost all markers tested here were elevated in the patient group. Other pathophysiological features play an important role as well. For instance, severe malarial anaemia can be caused by haemolysis, indicated by elevated lactate dehydrogenase, and bone marrow suppression [11].

Of the inflammatory markers tested, CRP showed the highest sensitivity and specificity in the malaria patients. The test is inexpensive and widely available in health institutions. However, the cut-off value of 10.8 mg/l is relatively low and can be found in many conditions, indicating mild inflammation and a challenge to the immune system, as seen in smokers [12]. It is not specific for P. falciparum infection. In sepsis, for example, elevated CRP correlates with disease severity [13,14]. Copeptin is known to be elevated in patients with sepsis [15] and myocardial damage [16,17]. In the malaria patients, significantly elevated levels but no association regarding the severity of malaria were found. This in line with previous findings [18]. PAPP-A was not significantly elevated in the patient group. In order to assure unambiguous results, patients with other co-existing infections were excluded from the study.

Furthermore, parameters related to endothelial activity were assessed. Endothelial damage can cause vascular leakage. In combination with microvessel obstruction due to microcirculatory disturbance, this can lead to cerebral involvement [19]. ICAM-1 and VCAM-1 play a major role as receptors on endothelial cells for P. falciparum-infected erythrocytes, indicating vascular injury [20]. In cerebral malaria correlation of increased local cellular expression of ICAM-1 with sequestration of infected parasites in the brain has been well demonstrated [21]. Measurement of sICAM-1 is not useful in severe malaria, characterized by malarial anaemia. This indicates that the question of whether or not markers are specific for disease manifestation, like severe malarial anaemia or cerebral malaria, can only be answered by conducting larger studies with a sufficient number of cases in respective subgroups of severe malaria. Myeolperoxidase is stored in and released by activated neutrophilic granulocytes in inflammatory conditions. Elevated levels can be found in patients with, myocardial or endothelial damage [22,23], and in inflammatory and infectious diseases such as sepsis, brucellosis and HIV [24-26]. Very high levels were found in the malaria group, reflecting vascular damage and inflammation. The protease elastase-2 is released by activated neutrophilic granulocytes, the vasoconstrictor endothelin-1 by activated endothelial cells. Elevated elastase-2 levels can be found in inflammatory conditions such as systemic inflammatory response syndrome [27,28]. Both parameters were significantly elevated in the malaria group. As described before, further analysis of prognostic qualities confirmed low discriminative power [29,30].

The exact mechanism of impaired cardiac function in P. falciparum malaria is still not fully understood [31,32]. NT-proBNP and MR-proANP, are sensitive markers for left ventricular dysfunction. In this study, measurements showed a significant difference between the controls and the patient group.

Coagulopathy may contribute to both, significant thrombocytopaenia, as shown in the patient group, as well as microcirculatory dysfunction. Increased fibrinolytic activity and coagulopathy in the malaria patients was reflected by significantly elevated levels of fibrinogen, and D-Dimers. None of the markers tested positive for prognostic qualities.

In non-endemic countries like Germany, the incidence of imported P. falciparum malaria is low, and many physicians lack experience dealing with the disease. Diagnosis and treatment are often delayed, potentially leading to complications with an increased mortality. In order to improve risk stratification at first presentation of malaria patients, it would be helpful to support standard diagnostic microscopy with additional diagnostic and prognostic biomarkers in in- and outpatient settings. Most markers tested are well suited to differentiate between P. falciparum malaria and healthy controls. In particular, in cases indicative for malaria due to symptoms and travel history, elevated levels may be useful to uphold the suspective diagnosis of P. falciparum infection. In these cases, parasitological confirmation of the diagnosis should be performed timely.

The limited number of patients examined in this prospective pilot study as well as the unmatched case control design increases the risk of type I errors. In order to assess the heterogenic pathophysiology of P. falciparum infection further, higher patient numbers categorized in different patient subgroups according to WHO criteria for severe malaria would have been favourable. It is possible that pathophysiological processes differ amongst travellers with different ethnic backgrounds. The study population examined here was quite heterogenous, and a matched control group would have been favourable.

## Conclusion

Several biomarkers tested in this study have been significantly associated with malaria, however the practical relevance remains to be proven. No practical relevance for discrimination between complicated and uncomplicated malaria was found in this pilot study.

## Competing interests

The authors declare that they have no competing interests.

## Authors’ contributions

SST and JPC drafted the manuscript. JPC and GDB conceived of the study, and participated in its design and coordination. NWB and SB carried out several immunoassays. JH carried out recruitment of study subjects. AH participated in the design of the study and performed the statistical analysis. All authors read and approved the final manuscript.

## Supplementary Material

Additional file 1 **Complicated malaria according to national guidelines **[8]**, and modified WHO criteria **[9]**.** Click here for file

Additional file 2 Biomarkers in all malaria patients versus controls, measured within 24 hours after first presentation. Click here for file

Additional file 3 Concentration of inflammatory, endothelial, cardiac, coagulation, and haemolysis markers in all malaria patients, uncomplicated malaria (UM), complicated malaria (CM), and healthy controls. Click here for file
