# Supplementary material for: Clinical relevance of different biomarkers in imported plasmodium falciparum malaria in adults: a case control study
Source: Malar J. 2013 Jul 16;12:246. doi: 10.1186/1475-2875-12-246 (PMC3724717; doi:10.1186/1475-2875-12-246)
Supplement: Additional file 1 — Complicated malaria according to national guidelines [8], and modified WHO criteria [9]. [file 1475-2875-12-246-S1.doc]

**Additional file 1:**

**Complicated malaria according to national guidelines [8], and modified WHO criteria [9]**

| **Clinical features** | **Laboratory findings** |
| --- | --- |
| Impaired level of consciousness or coma  Cerebral convulsions  Acidotic breathing, respiratory distress  Hypotension, circulatory collapse  (systolic blood pressure < 70mmHg)  Jaundice  Abnormal spontaneous bleeding  Pulmonary oedema | Hypoglycaemia  (blood glucose < 40 mg/dl)  Metabolic acidosis  (plasma bicarbonate < 15mmol/l)  Severe anaemia (Hb < 80g/l)  Haemoglobinuria  Hyperparasitaemia  (> 5%)  Renal impairment (serum creatinine > 2.5 mg/dl) |
