# Supplementary material for: Clinical relevance of different biomarkers in imported plasmodium falciparum malaria in adults: a case control study
Source: Malar J. 2013 Jul 16;12:246. doi: 10.1186/1475-2875-12-246 (PMC3724717; doi:10.1186/1475-2875-12-246)
Supplement: Additional file 2 — Biomarkers in all malaria patients versus controls, measured within 24 hours after first presentation. [file 1475-2875-12-246-S2.doc]

| **Additional file 2: Biomarkers in all malaria patients *versus* controls, measured within 24 hours after first presentation** | **Case /**  **Control** | **Number**  **(n)** | **Median**  **value** | **AUROC** | **95% CI** | | **Sensitivity** | **Specificity** | **Youden’s**  **Index** | **Cut-off**  **value** | **PPV**  **(%)**** | **NPV**  **(%)***** | **P-value*** |
| --- | --- | --- | --- | --- | --- | --- | --- | --- | --- | --- | --- | --- | --- |
|  |
| **INFLAMMATORY MARKERS** | | |  |  |  |  |  |  |  |  |  |  |  |
| PAPP-A (IU/l) | Case | 79 | 6.0 | 0.494 | 0.386 | 0.602 | 0.09 | 1.00 | 0.09 | 11.0 | 100.0 | 36.3 | 0.912 |
|  | Control | 41 | 6.3 |
| Copeptin (pmol/l) | Case | 79 | 8.9 | 0.812 | 0.735 | 0.888 | 0.76 | 0.78 | 0.53 | 5.7 | 87.0 | 62.0 | < 0.001 |
|  | Control | 40 | 4.5 |
| CRP (mg/l) | Case | 79 | 90.0 | 1000 | 1000 | 1000 | 1.00 | 1.00 | 1.00 | 10.8 | 100.0 | 100.0 | < 0.001 |
|  | Control | 39 | 5.0 |
| **ENDOTHELIAL MARKERS** | |  |  |  |  |  |  |  |  |  |  |  |  |
| MPO (pmol/l) | Case | 79 | 3110.4 | 0.989 | 0.975 | 1000 | 0.09 | 1.00 | 0.09 | 8938.8 | 100.0 | 36.3 | < 0.001 |
|  | Control | 41 | 527.0 |
| Elastase-2 (ng/ml) | Case | 79 | 2959.0 | 0.979 | 0.956 | 1000 | 0.95 | 0.90 | 0.85 | 747.5 | 94.9 | 90.2 | < 0.001 |
|  | Control | 41 | 207.0 |
| Endothelin-1 (fmol/ml) | Case | 79 | 1.2 | 0.741 | 0.651 | 0.832 | 0.47 | 0.90 | 0.37 | 1.4 | 88.1 | 46,2 | < 0.001 |
|  | Control | 41 | 0.6 |
| sICAM-1 (ng/ml) | Case | 79 | 142.3 | 0.977 | 0.955 | 0.999 | 0.92 | 1.00 | 0.92 | 56.2 | 100.0 | 84.6 | < 0.001 |
|  | Control | 41 | 17.1 |
| sVCAM-1 (ng/ml) | Case | 79 | 1120.0 | 0.911 | 0.858 | 0.964 | 0.77 | 0.93 | 0.70 | 667.5 | 95.3 | 67.9 | < 0.001 |
|  | Control | 41 | 420.0 |
| **CARDIAC MARKERS** | |  |  |  |  |  |  |  |  |  |  |  |  |
| NT-proBNP (pg/ml) | Case | 79 | 61.9 | 0.695 | 0.602 | 0.789 | 0.43 | 0.93 | 0.36 | 86.2 | 91.9 | 45.8 | < 0.001 |
|  | Control | 41 | 35.6 |
| MR-proANP (pmol/l) | Case | 79 | 44.3 | 0.721 | 0.632 | 0.810 | 0.48 | 0.93 | 0.41 | 46.1 | 92.7 | 47.4 | < 0.001 |
|  | Control | 40 | 28.3 |
| **COAGULATION MARKERS** | |  |  |  |  |  |  |  |  |  |  |  |  |
| Fibrinogen (g/l) | Case | 71 | 5.0 | 0.910 | 0.852 | 0.924 | 0.92 | 0.85 | 0.76 | 3.2 | 90.3 | 84.2 | < 0.001 |
|  | Control | 39 | 2.7 |
| D-Dimers (mg/l) | Case | 58 | 3.9 | 0.980 | 0.958 | 0.924 | 0.86 | 1.00 | 0.86 | 0.6 | 98.0 | 82.6 | < 0.001 |
|  | Control | 39 | 0.3 |
| Platelets (x1000/µl) | Case | 79 | 85.0 | 0.958 | 0.922 | 0.995 | 0.87 | 0.97 | 0.85 | 163.0 | 83.3 | 97.0 | < 0.001 |
|  | Control | 39 | 236.0 |
| **HAEMOLYSIS MARKERS** | |  |  |  |  |  |  |  |  |  |  |  |  |
| LDH (U/l) | Case | 77 | 273.0 | 0.967 | 0.936 | 0.924 | 0.90 | 0.97 | 0.87 | 196.5 | 98.6 | 82.6 | < 0.001 |
|  | Control | 39 | 152.0 |

95% CI = 95% Confidence Interval for AUROC; *P-value for AUROC; **PPV: positive predictive value; ***NPV: negative predictive value
