# Supplementary material for: Clinical relevance of different biomarkers in imported plasmodium falciparum malaria in adults: a case control study
Source: Malar J. 2013 Jul 16;12:246. doi: 10.1186/1475-2875-12-246 (PMC3724717; doi:10.1186/1475-2875-12-246)
Supplement: Additional file 3 — Concentration of inflammatory, endothelial, cardiac, coagulation, and haemolysis markers in all malaria patients, uncomplicated malaria (UM), complicated malaria (CM), and healthy controls. [file 1475-2875-12-246-S3.doc]

**Additional file 3** Concentration of inflammatory, endothelial, cardiac, coagulation, and haemolysis markers in all malaria patients, uncomplicated malaria (UM), complicated malaria (CM), and healthy controls

**___________________________________________________________________________**

**INFLAMMATORY MARKERS**


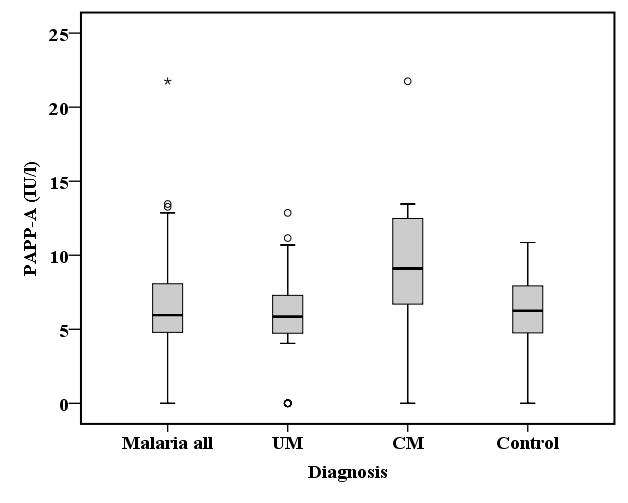

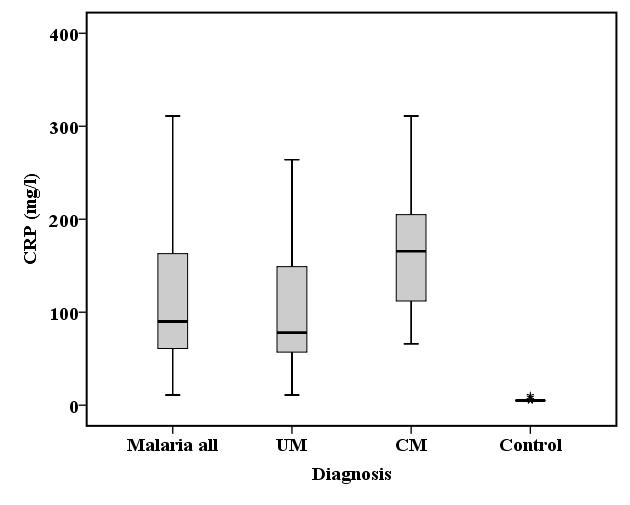

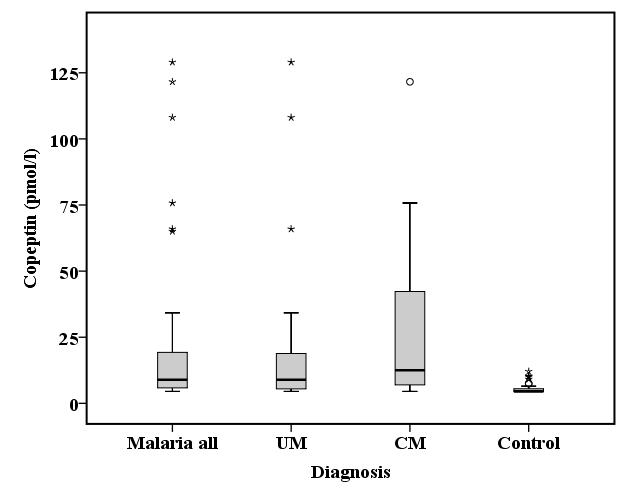


**ENDOTHELIAL MARKERS**


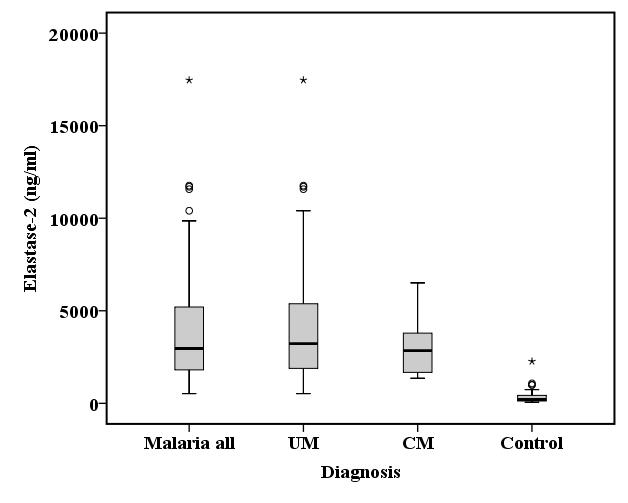

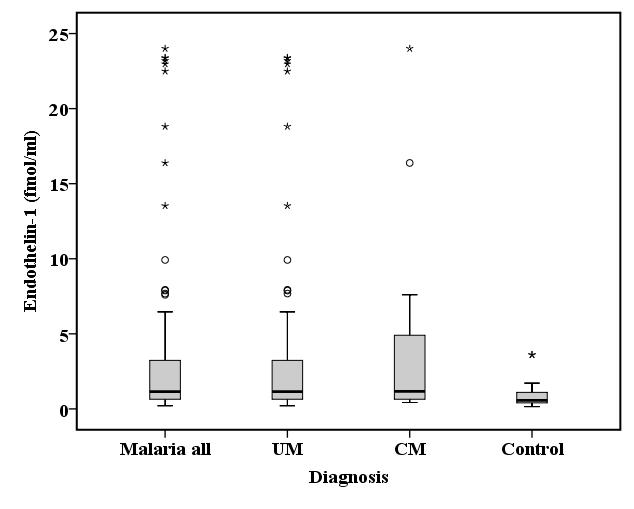

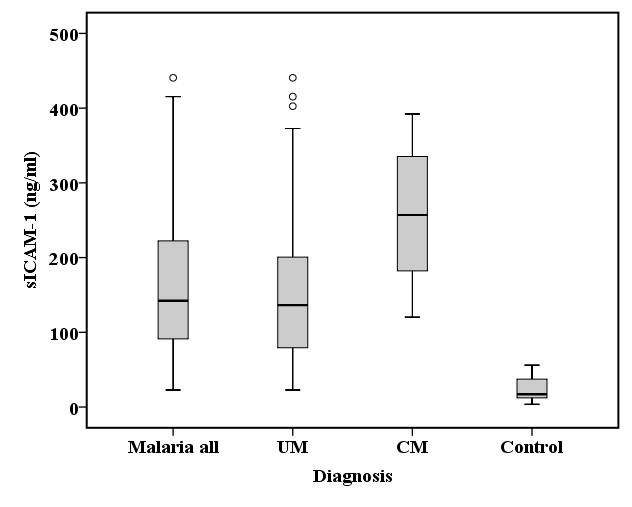

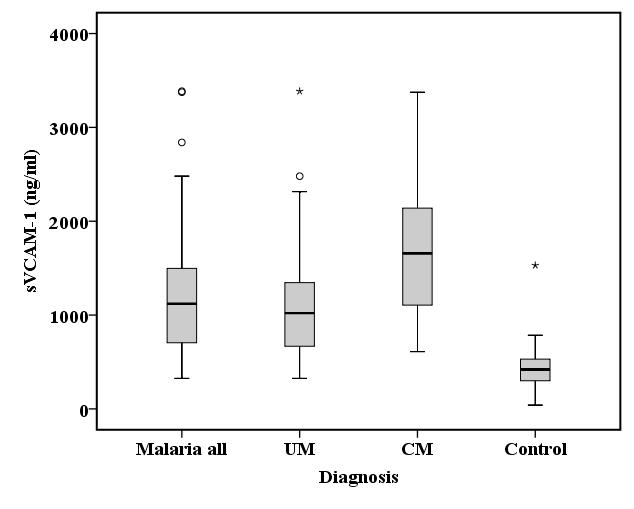

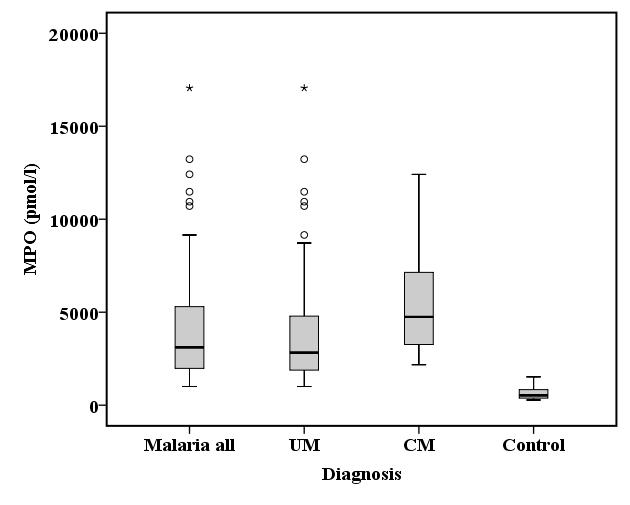


**CARDIAC MARKERS**


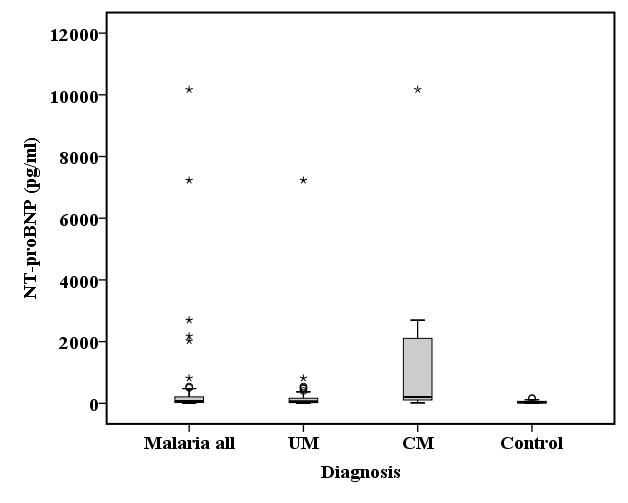

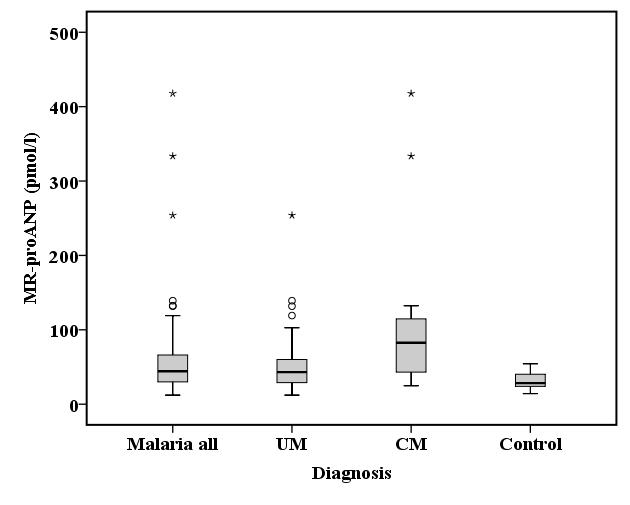


**COAGULATION MARKERS**


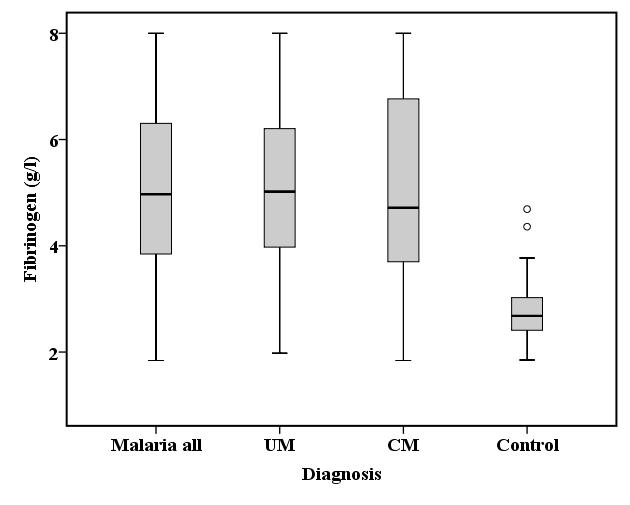

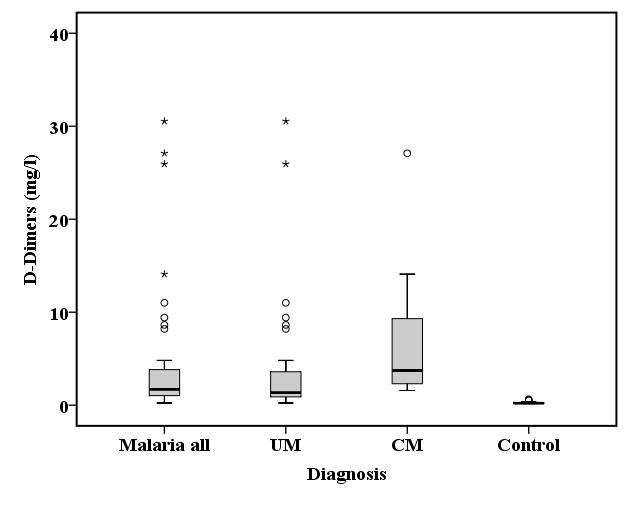

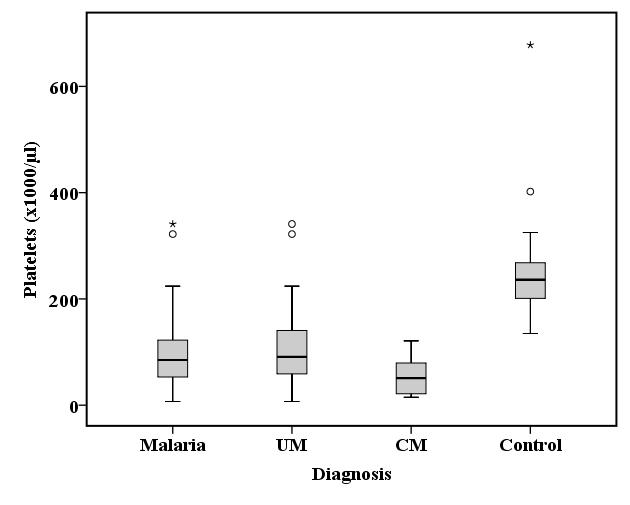

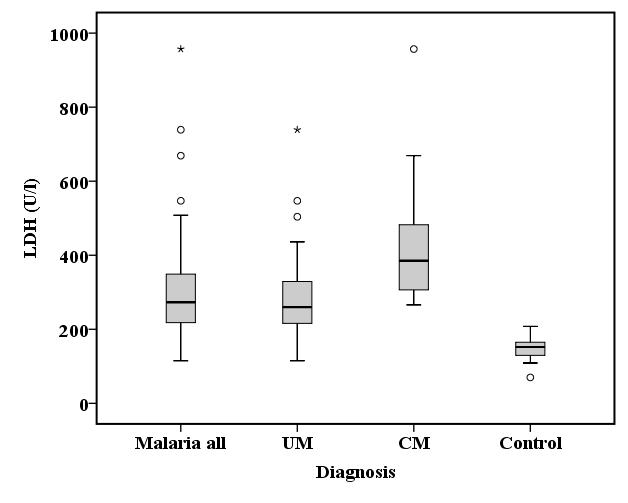


**HAEMOLYSIS MARKER**

___________________________________________________________________________
